# Association Between Ankylosing Spondylitis and Periodontitis: A Systematic Review

**DOI:** 10.3290/j.ohpd.c_2747

**Published:** 2026-06-25

**Authors:** Valentin Quemard, Zocko Ange Désiré Pockpa, Christian Verner, Assem Soueidan, Camille Bechina, Xavier Struillou

**Affiliations:** a Valentin Quemard Assistant, Department of Periodontology, Faculty of Dental Surgery, Nantes Université, Nantes, France. Idea, contributed to the conception, study design, data collection, data interpretation, and wrote the manuscript.; b Zocko Ange Désiré Pockpa Assistant, Department of Periodontology, Faculty of Dental Surgery, University of Felix Houphouët Boigny, Abidjan, Ivory Coast, West Africa. Contributed to the interpretation of the data, read and approved the manuscript.; c Christian Verner Associate Professor of Periodontology, Department of Periodontology, Faculty of Dental Surgery, Nantes Université, Nantes, France. Contributed to critical proofreading and discussion, read and approved the manuscript.; d Assem Soueidan Professor of Periodontology, CHU Nantes, Nantes Université, Periodontology Department, UIC Odontology 11, Nantes, France; Nantes Université, CHU Nantes, INSERM, Regenerative Medicine and Skeleton, RMeS, Nantes, France. Contributed to critical proofreading and discussion, read and approved the manuscript.; e Camille Bechina Assistant, Department of Periodontology, Faculty of Dental Surgery, Nantes Université, Nantes, France. Study design, data collection, data interpretation, read and approved the manuscript.; f Xavier Struillou Associate Professor of Periodontology, CHU Nantes, Nantes Université, Periodontology Department, UIC Odontology 11, Nantes, France; Nantes Université, CHU Nantes, INSERM, Regenerative Medicine and Skeleton, RMeS, Nantes, France. Contributed to the conception, acquisition, analysis or interpretation of the data, read and approved the manuscript.

**Keywords:** ankylosing spondylitis, periodontitis, periodontal diseases, spondyloarthritis, systematic review

## Abstract

**Purpose:**

This systematic review aimed to investigate a potential association between ankylosing spondylitis (AS) and periodontitis (PD), two chronic inflammatory diseases.

**Methods and Materials:**

Following the Preferred Reporting Items for Systematic Reviews and Meta-Analyses (PRISMA) 2020 guidelines, searches were conducted in PubMed, The Cochrane Library, Scopus, and Web of Science. Keywords included ‘spondyloarthritis’, ‘ankylosing spondylitis’, ‘periodontal diseases’, and ‘periodontitis’. Study selection was performed by independent authors, and quality was assessed using Scottish Intercollegiate Guidelines Network (SIGN) methodology and the STROBE-MR statement.

**Results:**

Out of 255 initial studies, 18 eligible studies (3 cohort, 14 case-control, 1 Mendelian randomisation) were selected, all of acceptable to high quality. The prevalence of PD in AS patients showed conflicting results. Periodontal parameters like bleeding on probing (BOP), probing pocket depth (PPD), and clinical attachment loss (CAL) didn’t consistently show statistically significant differences but were frequently correlated with AS parameters (eg, MASES, BASDAI, BASFI, CRP, AS duration). AS treatment with anti-TNF-α agents was associated with a decrease in PPD and CAL. Other findings included an increase in Gingival Index in AS patients treated with anti-TNF-α and mixed results for the Decayed, Missing, and Filled Teeth Index. Microbiological studies showed inconsistent results for *Porphyromonas gingivalis* (Pg), but anti-Pg IgG levels correlated with AS parameters. PD appeared to significantly increase inflammatory markers (IL-6, TNF-α, CRP, ESR) in AS patients. Only one study investigated a genetic association, finding none.

**Conclusion:**

A definitive conclusion regarding an increased prevalence of PD in AS patients cannot be drawn with certainty from this review, possibly due to heterogeneous classification criteria. However, the correlation between PD and AS parameters, alongside the impact of AS therapy on periodontal health, is noteworthy. Screening and treating periodontal disease in AS patients might delay symptom progression and reduce drug dosage, though targeted clinical trials are necessary to confirm these findings.

Ankylosing spondylitis (AS) is a progressive, chronic, inflammatory rheumatic disease affecting the axial skeleton’s joints. Its prevalence varies from 0.1% to 0.2% depending on the population. Its development is multifactorial but appears primarily genetic through the involvement of immune pathways. Among these immune pathways, interleukins, as well as CD8 and CD4 T lymphocytes, are involved. Thus, even if the human leukocyte antigen B27 (HLA-B27) antigen is found in only 8% of the general population, it is present in 90% to 95% of European Caucasian patients with AS.^26,40^

The current classification used for ankylosing spondylitis is the one proposed by the Assessment of SpondyloArthritis International Society (ASAS) in 2009.^38^ The establishment of a diagnosis is predicated on the concomitant presence of at least one of the three following clinical criteria: inflammatory low back pain of ≥ 3 months’ duration (characterised by improvement with activity and lack of attenuation at rest), restricted lumbar range of motion (sagittal and frontal planes), and/or reduced chest expansion (relative to age- and sex-specific normative values). This clinical presentation must be combined with one of the radiographic criteria: the detection of bilateral sacroiliitis of grade ≥ 2 or unilateral sacroiliitis of grade ≥ 3. These radiographs are significant, as the progression of this pathology appears to be correlated with a radiographic evolution.^15,26^

Periodontitis (PD) is also a chronic, multifactorial inflammatory disease resulting from dysbiosis.^22^ Locally, it leads to the irreversible loss of part or all of the tooth’s supporting tissues. Clinical symptoms can include attachment loss due to the presence of periodontal pockets and/or recessions, with or without alveolar bone loss, all of which can lead to tooth mobility or tooth loss. However, it can also manifest as gingival bleeding, indicating gingival inflammation.^23^ Furthermore, there has been long-standing evidence that periodontitis exerts a systemic impact, influencing various conditions including autoimmune disorders, metabolic syndromes, pregnancy complications, neurodegenerative diseases, cardiovascular pathologies, and viral infections.^18^ Consensus reports have shown that treating periodontitis can improve health outcomes for patients with diabetes, leading to a decrease in their HbA1c levels, and for those with cardiovascular diseases, by positively affecting blood pressure and other markers.^6,32^

The systemic actions of periodontitis are thought to be due to the development of low-grade systemic inflammation. This is characterised by a baseline increase in pro-inflammatory molecules like interleukin-6 (IL-6) and CRP. Moreover, pathogenic periodontal bacteria like *Porphyromonas gingivalis* (Pg) might indirectly trigger the release of IL-39, which could subsequently influence ankylosing spondylitis.^2^ This increase could make patients more susceptible to other inflammatory diseases. It also seems that periodontitis has an effect on systemic bone resorption. Specifically, the blood mononuclear cells of patients with periodontal disease undergo an accelerated osteoclastic transformation, which can worsen certain bone pathologies. This low-grade systemic inflammation and increased myelopoietic activity are thought to be promoted by bacteraemia or spillover of inflammatory mediators from periodontal tissues into the bloodstream.^13,42^

As previously noted, periodontitis is now recognised as a distinct systemic condition influenced by diverse risk factors, including lifestyle and genetic profiles.^41^ It exhibits bidirectional interactions – both local and systemic – with other pathologies, primarily mediated by cytokines such as IL-1, IL-6, and TNF-α.^2,41^ These cytokines play a pivotal role in regulating inflammation and tissue degradation, particularly within the periodontium. Consequently, treating systemic diseases may impact periodontitis or its management by modulating microbial agents or the host response.^9^ In the future, these inflammatory molecules could serve as biomarkers to assess periodontal disease severity, treatment outcomes, and the collateral impact of systemic therapies on periodontal tissues.^10^

The aim of this study is to investigate the multifaceted links between ankylosing spondylitis (AS) and periodontal disease (PD), while evaluating the reciprocal effects of their respective treatments on both pathologies.

Thus, PD can be induced or aggravated by systemic pathologies. Indeed, the 2017 Chicago classification highlights three main categories of systemic disorders or pathologies influencing periodontal disease: systemic disorders with a major impact on periodontal tissue loss by influencing periodontal inflammation, other systemic disorders that influence the pathogenesis of periodontal diseases, and systemic disorders that can lead to periodontal tissue loss independently of PD.^19^ In the second category, we find arthritis, notably rheumatoid arthritis.^19^ Since this pathology is similar in some respects to AS, it seems legitimate to seek a link between PD and the latter. Furthermore, we determined that for cardiovascular diseases (CVDs), periodontal treatment can reduce CRP. Now, CRP is a molecule that is also studied in rheumatic diseases, particularly rheumatoid arthritis and AS. The aim of this study is to investigate whether a link exists between AS and PD, given their potential shared inflammatory relationship.

## METHODS AND MATERIALS

For this research, we adhered to the guidelines set forth by the 2020 Preferred Reporting Items for Systematic Reviews and Meta-Analyses (PRISMA) statement.^28^ This review was registered in the Prospective International Registry of Systematic Reviews (PROSPERO) under ID CRD42024599011. The aim of this study is to investigate the multifaceted links between ankylosing spondylitis (AS) and periodontal disease (PD), while evaluating the reciprocal effects of their respective treatments on both pathologies.

The PICOS structure was used to define the inclusion criteria and formulate the research question. Studies that met the following criteria were included:

P – Population/Patients:

Patients diagnosed with AS, with different stages and severities of the disease

I – Intervention/Exposure:

Presence or severity of PD
Exposure is defined by clinical parameters including: PPD, CAL, BOP and PI

C – Comparison: The presence and characteristics of PD in AS patients compared to:

AS patients without PD
Healthy controls (individuals without AS or PD)
Patients with another rheumatic pathology
Controls with PD but without AS

O – Outcomes:

Prevalence and risk of PD in AS patients compared to control groups
AS disease activity: BASDAI, ASDAS, BASFI, mSASSS and BASMI
Systemic inflammation: ESR and CRP
Oral health status: DMFT, PISA, and microbiome composition
Genetic association: links through SNPs

S – Study design:

Randomised controlled trial
Cohort studies
Case-control studies
Mendelian randomisation analysis
Cross-sectional studies

### Search Strategy

We conducted searches across the following four databases: PubMed, The Cochrane Library, Scopus and Web of Science. A specific search equation was formulated for each database, utilising the following keywords and MeSH terms: spondyloarthritis, ankylosing spondylitis, periodontal diseases, and periodontitis. Table 1 presents the search strategies for each database. Our selection included studies published from January 2000 through September 2025. Lastly, a manual bibliographic search was also conducted.

**Table 1:** Databases and search terms


Pubmed (Filtrer: MEDLINE)	((Spondyloarthritis) OR (ankylosing spondylitis) OR (ankylosing spondylitis[MeSH Terms]) OR (Spondyloarthritis[MeSH Terms])) AND ((Periodontitis[MeSH Terms]) or (Periodontal Diseases[MeSH Terms]) or (Periodontal Diseases or Periodontitis))
Cochrane library (All text)	Spondyloarthritis OR ankylosing and spondylitis AND Periodontitis OR Periodontal and Diseases
Scopus (Article title, Abstract, Keywords)	ankylosing and spondylitis OR Spondyloarthritis AND Periodontitis OR Periodontal and Diseases
Web of Science (Search: MEDLINE; Editions: All; All Fields)	Spondyloarthritis OR ankylosing and spondylitis AND Periodontitis OR Periodontal and Diseases


### Article Selection and Data Extraction

Titles, abstracts, and full texts were screened independently by VQ and CB. Any disagreements were resolved by consensus or, if necessary, adjudicated by a third independent reviewer (WS). This third reviewer was blinded to the specific opinions and reasons for disagreement of the first two authors, ensuring an impartial and independent assessment for the final inclusion decision. A table was previously established to provide support for the collection of the following data (Table 2).

**Table 2:** Characteristics of the selected studies

Author, year, Country, reference	Study type	Study duration (week)	Sample size	Age Mean (±SD) (years)	Gender (male/female)	Duration of AS (years; mean ± SD)	Diagnostic criteria used for AS	Diagnostic criteria used for PD	PD parameter studied	AS parameter studied	Others parameters studied
Helenius et al, 2005, Finland^16^	Case control	NA	Cases: 18 Controls: 18	Cases: 42.4 (± 11.6) Controls: 43.4 (± 12.2)	Cases: 12/6 Controls: 12/6	Cases: 14.7 ± 8.9	Modified New York criteria	CPITN	Dental calculus, PPD, BOP, periodontal status	NA	Manior salivary gland biopsy, salivary biochemical analyses, oral mucosal diseases,signs of dental infection foci, caries lesions
Sezer et al, 2011, Turkey^36^	Case control	NA	Cases: 48 Controls: 48	Cases: 34.27 (± 9.73) Controls: 33.33 (± 9.67)	Cases: 35/13 Controls: 35/13	Cases: 5.04 ± 6.13	Modified New York criteria	AAP 1999 guidelines	PI, PPD, CAL, BOP	BASDAI	CRP, ESR, TNF-α, IL-6, venous blood
Keller et al, 2013, Taiwan^21^	Case control	NA	Cases: 6,821 Controls: 34,105	NA	Cases: 3 982/2 839 Controls: 19 910/14 195	NA	Clinical symptoms or Radiography of the spine or HLA-B27 test	PPD ≥ 3 mm	PD prevelance	NA	NA
Kang et al, 2015, South Korea^20^	Case control	NA	Cases: 84 Controls: 84	Cases: 37 Controls: 36.5	Cases: 3 982/2 839 Controls: 19 910/14 195	Cases: 10.7	Modified New York criteria	Page and Eke CDC, Lopez et al, Pishon et al	PPD, CAL, PI, BOP	BASDAI, ASDAS, BASMI, chest expansion, mSASSS	DMFT, serum anti-Pg, Pg DNA, ESR, CRP
Fabri et al, 2015, Brazil^12^	Case control	26 (6 month)	Cases: 15 Controls: 15	Cases: 39.9 (± 12.9) Controls: 48.6 (± 11.6)	NA	NA	Modified New York criteria	NA	PPD, CAL, GBI, PI	BASDAI, BASFI, BASMI	CRP, ESR, treatment profile
Bisanz et al, 2016, New Zealand^5^	Case control	NA	Cases: 17 Controls: 22	Cases: 38 (± 12.8) Controls: 37 (± 12.7)	Cases:11/6 Controls: 15/7	NA	Modified New York criteria	Page and Eke 2007 CDC	PPD, gingival recession, CAL, BOP	BASDAI	Bacterial populations, Oral mucosal conditions, DMFT
Mohammed et al, 2017, Iraq^27^	Case control	NA	Cases: 45 Controls: 45	Cases: 37.7 (± 8.5) Controls: 32.4 (± 7.6)	Cases: 40/5 Controls: 25/20	Cases: 9.44 ± 7.1	NA	NA	PI, PPD, CAL	NA	NA
Bautista-Molano et al, 2017, Colombia^3^	Case control	NA	Cases: 78 Controls: 156	Cases: 39.6 (± 11) Controls: 39.5 (± 11.1)	Cases: 47/31 Controls: 94/62	NA	ASAS classification criteria	Page and Eke 2007 CDC	CAL, PPD, PI, GI	ASDAS-CRP	Pg antibodies (IgG1, IgG2), Td, Tf, Pg
Iordache et al, 2017, Romania^17^	Cohort	24	Cases: 86	Baseline: 37.74 ± 18.33	NA	Cases: 34.18 ± 17.96	Modified New York criteria	AAP 1999 guidelines	PI, GI, BOP, PPD, CAL	BASDAI, ASDAS-CRP	ESR, CRP
Ziebolz et al, 2018, Germany^45^	Case control	NA	Cases: 52 Controls: 53	Cases: 49.5 Controls: 48	Cases: 28/24 Controls: 28/25	NA	NA	Page and Eke 2007 CDC	Papillary bleeding index, PPD, CAL	NA	DMF-T, Microbiological analysis of 11 periodontal pathogenic bacteria
Schmalz et al, 2018, Germany^34^	Case control	NA	Cases: 50 Controls: 50	Cases: 47.18 ± 15.67 Controls: 55.82 ± 10.56	Cases: 26/24 Controls: 23/27	Cases: 10.92 ± 10.55	Modified New York criteria	AAP 1999 guidelines	Periodontal conditions (diagnostic)	BASDAI, BASMI, BASFI, BASG, swollen joints, painful joints, morning stiffness,	DMF-T, OHIP-G14
Białowąs et al, 2019, Poland^4^	Case control	NA	Cases: 30 Controls: 25	Cases: 43 Controls: 45^1y^	Cases: 26/4 Controls: 20/5	Cases: 12 ± 8	ASAS 2009/2011 criteria	Offenbacher classification	PI, BOP, PPD	NA	Number of teeth, Pg DNA, CRP, ESR, TNF-α, MMP-3, MMP-9
Enginar et al, 2021, Turkey^11^	Case control	NA	Cases: 129 Controls: 71	Cases: 40.12 ± 10.185 Controls: 39.39 ± 10.187	Cases: 102/27 Controls: 41/30	Cases: 15.3566 ± 831302 Controls: 11.3563 ± 8.75073	Modified New York criteria	AAP 1999 guidelines	PI, GI, PPD, CAL, BOP	BASDAI, BASMI, BASFI, MASES, ASQoL scale	ESR, CRP
Schmalz et al, 2021, Germany^33^	Case control	NA	Cases: 32 Controls: 101	Cases: 48.37 ± 16.77 Controls: 57.48 ± 10.03	Cases: 18/14 Controls: 19/82	Cases: 11.00 ± 12.18 Controls: 6.60 ± 8.49	Modified New York criteria	2017 World Workshop criteria	PISA, CAL, PPD, BOP	BASDAI, BASMI, BASFI, BASG, morning stiffness	CRP, HLA-B27+, number of teeth
Daltaban et al, 2023, Germany^8^	Case control	NA	Cases: 28 Controls: 28	Cases: 39.93 ± 11.29 Controls: 40.04 ± 10.91	Cases: 20/8 Controls: 19/9	Cases: 6.52 ± 6.78	Modified New York criteria	2017 World Workshop criteria	PI, BOP, PPD, CAL	BASDAI, MASES, BASMI, BASFI, morning siffness	Gingival crevicular fluid level of sclerostin-IL1β /MMP8, CRP, drugs used in AS
Han et al, 2024, China^14^	Mendelian randomisation analysis	AS Cases: 968 AS Controls: 336,191 PD Cases: 17,353 PD Controls: 28,210	NA	NA	NA	NA	Genetic association from the Neale Laboratory Consortium	Genetic association from the Gene-Lifestyle Interactions and Dental Endpoints (GLIDE)	NA	NA	80 independent SNPs for PD, 39 independent SNPs for AS
Chung et al, 2024, South Korea^7^	Cohort	52	Baseline: 2 271 221	Baseline: 42.14 ± 12.75	Baseline: 1 505 305 /765 916	NA	Modified New York criteria	Having two claims for periodontitis diagnosis codes and at least one treatment code or periodontal pocket detected by a dentist during examination	Dental scaling, oral hygiene bihaviors	NA	Body mass index, smoking status, alcohol consomption, regular physical activity, comorbidities
Yun et al, 2024, South Korea^43^	Cohort	208 (4years)	Baseline: 489 125	Baseline: 56.4 ± 8.1	Baseline: 265 195 / 223 929	NA	NA	Self reported examination	Self reported oral health was used (bleeding, painful gums and losse teeth)	NA	NA
AS: ankylosing spondylitis, ASAS: Assessment of SpondyloArthritis International Society, ASDAS: Ankylosing Spondylitis Disease Activity Score, BASDAI: Bath Ankylosing Spondylitis Disease Activity Index, BASFI: Bath Ankylosing Spondylitis Functional Index, BASG: Bath Ankylosing Spondylitis Global Score, BASMI: Bath Ankylosing Spondylitis Metrology Index, BOP: bleeding on probing, CAL: clinical attachment loss, CDC: Centers for Disease Control and Prevention, CPITN: Community Periodontal Index of Treatment Needs, CRP: C-reactive protein, DMFT: decayed, missing, and filled teeth, ESR: erythrocyte sedimentation rate, GBI: Gingival Bleeding Index, HLA-B27: Human Leukocyte Antigen B27, mSASSS: modified Stoke Ankylosing Spondylitis Spine Score, PD: periodontal disease, Pg: *Porphyromonas gingivalis,* PI: Plaque Index, PISA: periodontal inflammatory surface area, PPD: pocket probing depth, Td: *Treponema denticola*, Tf: *Tannerella forsythia*, TNF-α: tumour necrosis factor alpha. ^y^ result given as median and not mean.											

### Quality Assessment

As no specific Cochrane recommendations were identified for bias analysis in observational studies, we used the Scottish Intercollegiate Guidelines Network (SIGN) methodology checklist, appropriate for the selected case-control and cohort study designs^35^ and the STROBE-MR statement for the Mendelian randomisation (MR) study.^39^

## RESULTS

**Fig 1:**
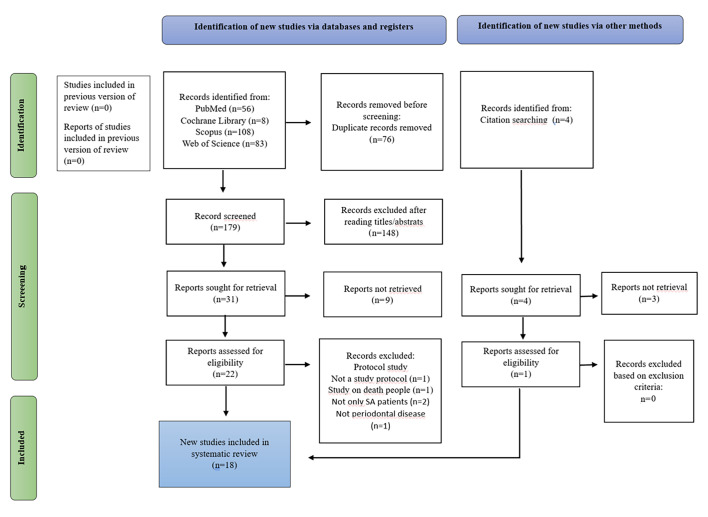
Flowchart.

Following our search, 255 studies were initially retrieved (Fig 1). After removing 76 duplicates with ZOTERO, 148 studies were excluded after screening titles and abstracts. This left 31 articles, but 9 could not be retrieved in full text, preventing proper evaluation of their ‘Materials and Methods’ section and leading to their exclusion.

An additional 5 studies were excluded due to reasons such as ‘Not a study protocol’, ‘Study on dead people’, ‘Not only AS patients’, and ‘Not periodontal disease’. Consequently, our database search yielded 17 eligible studies. A manual bibliographic search identified 4 additional studies; however, 3 were excluded as only their abstracts were available. In total, 18 articles met the inclusion criteria and were included in the systematic review.

These 18 studies consisted of three different designs: 3 cohort studies^7,17,43^ and 14 case-control studies^3–5,8,11,12,16,20,21,27,33,34,36,45^ and 1 MR analysis study.^14^ The cohort studies, published in 2017 and 2024, enrolled 86 to 2,271,221 patients over a duration of 24 weeks to 1 year, and were conducted in Romania and Korea. The case-control studies were published between 2005 and 2023, including 15 to 200 participants from 10 different countries. The single MR study was published in 2024 in China. An overview of study characteristics and findings can be found in Table 2.

### Periodontal Parameters

Six periodontal parameters were studied: bleeding on probing (BOP); probing pocket depth (PPD); clinical attachment loss (CAL); periodontal inflamed surface area (PISA); plaque index (PI); and gingival index (GI). Results for these parameters were also mixed.

For BOP, three studies found a statistically significant increase in AS patients,^11,20,33^ while two others found no significant difference.^5,8^ A decrease in BOP was observed in one study and after anti-TNF treatment.^17^ Finally, numerous correlations have been found between BOP and AS parameters. It is correlated with Maastricht Ankylosing Spondylitis Enthesitis Score (MASES), Bath Ankylosing Spondylitis Disease Activity Index (BASDAI), Bath Ankylosing Spondylitis Functional Index (BASFI), Ankylosing Spondylitis Quality of Life (ASQoL), CRP, and Bath Ankylosing Spondylitis Metrology Index (BASMI).^8,34^

Regarding PPD, one study showed a significant increase,^45^ another reported a significant decrease,^33^ and three others found no significant differences.^3,12,17^ PPD was correlated with increased BASMI, AS duration, and CRP levels.^8,45^ AS treatment with anti-TNF-α was shown to decrease PPD.^3,12,17^

Similarly, while one study identified significantly higher CAL in AS patients,^45^ a second found a significant decrease.^33^ In contrast, three studies found no significant differences.^8,21,27^ Correlations between CAL and various AS parameters, as well as an inflammatory parameter, emerged from our selection of articles. In patients with AS, a correlation between CAL and IL-6 was identified.^20^ It would also be correlated with BASMI and AS duration, as well as BASMI and MASES.^8,45^ Like PPD, AS treatment with anti-TNF-α led to a decrease in CAL.^3,12,17^

Schmalz et al are the only ones who investigated PISA, finding no association with disease parameters.^33^

Enginar et al demonstrated a significant increase in GI in AS patients treated with anti-TNF-α, with a correlation to MASES.^11^

Finally, no significant difference was identified for the PI in three studies.^8,21,27^

### Prevalences

Findings on the prevalence of PD in AS patients are inconsistent. Two studies found no increased prevalence,^4,21^ while one even showed a decrease in prevalence and severity after anti-tumour necrosis factor (TNF) therapy.^3^ Schmalz et al noted that AS patients had a lower periodontal stage but a more frequent grade C.^33^ Conversely, four studies reported an increased PD prevalence in AS patients,^21,27,43,45^ and two studies also identified a correlation between increased AS prevalence and PD. The respective hazard ratios (HR) were 1.33 and 1.15.^7,43^

### Other Oral Health Parameters

Seven studies investigated the Decayed, Missing, and Filled Teeth Index (DMFT) or its components. Helenius et al found no significant difference in the number of carious or missing teeth between SA patients and the control group.^16^ This finding aligns with Bisanz et al, who also reported no significant difference in DMFT between SA patients and the control group.^5^

Conversely, Ziebolz et al observed a significant difference in the number of carious teeth within the AS patient group.^45^ Schmalz et al made the same observation, additionally noting a significant difference in the number of filled teeth in their first study and a decrease in the number of missing teeth in their second.^33,34^ In the latter study, they even identified a correlation between higher age and increased BASMI with a lower number of remaining teeth.^33^ Chun et al highlighted an increased occurrence of AS with an increased number of missing teeth.^7^

However, SA did not appear to impact the Community Periodontal Index of Treatment Needs (CPITN).^11,16^

One study investigated salivary modifications, observing a significant decrease in salivary secretion in the SA group, accompanied by an increase in salivary proteins and the presence of immunoglobulin-A (IgA).^16^ Moreover, one study showed a statistically significant decrease in crevicular fluid sclerostin in SA patients.^8^

Regarding oral hygiene habits, Kang et al’s study observed a significant decrease in the frequency of tooth brushing in the SA group.^20^ Improving oral hygiene habits and undergoing scaling and root planing within the year both reduced the occurrence of SA.^7^ Finally, Schmalz et al demonstrated an impaired oral health-related quality of life in SA patients using the Oral Health Impact Profile – German 14 (OHIP-G14).^34^

### Microbiology

Two studies reported no significant difference in Pg carrier rates, anti-Pg antibodies, or Pg DNA, respectively.^5,20^ A decrease in Pg, *Tannerella forsythia* (Tf), and *Treponema denticola* (Td) antibodies in the SA group was determined by Bautista-Molano et al.^3^ Additionally, an increase in *Prevotella intermedia* (Pi), *Enterococcus faecalis* (En), and *Escherichia coli* (Ec) in the SA group was evidenced by Ziebolz et al.^45^ Finally, Kang et al’s study demonstrated that anti-Pg IgG levels were positively correlated with modified Stoke Ankylosing Spondylitis Spinal Score (mSASSS) and BASMI and negatively correlated with chest expansion.^20^

### Inflammatory Markers and SA Activity

PD appeared to significantly increase inflammatory markers in SA patients. Specifically, a significant increase in IL-6, TNF-α, and CRP was observed in these patients.^20^ Chung et al identified that erythrocyte sedimentation rate (ESR) and CRP were significantly higher in patients suffering from both SA and PD compared to those with only SA.^7^

### Genetic Association

Only one study investigated a genetic association between PD and SA. However, with an odds ratio (OR) of 1, it showed no genetic association between these two pathologies.^14^

### Assessment of Bias Risk

As previously stated, we assessed the risk of bias in the studies using the SIGN methodology. This assessment required three distinct classifications: one for the three cohort studies (Table 3) and another for the 14 case-control studies (Table 4).^35^ Finally, for the MR study, we used the STROBE-MR methodology (Table 5).^39^

**Table 3:** Methodology checklist for cohort studies (SIGN)^35^

	Lordache et al, 2017^17^	Chung et al, 2024^7^	Yun et al, 2024^43^
1.1 The study addresses an appropriate and clearly focused question	Yes	Yes	Yes
1.2 The two groups being studied are selected from source populations that are comparable in all respects other than the factor under investigation	Yes	Yes	Yes
1.3 The study indicates how many of the people asked to take part did so, in each of the groups being studied	Does not apply	Yes	Does not apply
1.4 The likelihood that some eligible subjects might have the outcome at the time of enrolment is assessed and taken into account in the analysis	Does not apply	Can’t say	Does not apply
1.5 What percentage of individuals or clusters recruited into each arm of the study dropped out before the study was completed	Cannot say	0%	0%
1.6 Comparison is made between full participants and those lost to follow-up, by exposure status	Does not apply	Does not apply	Does not apply
1.7 The outcomes are clearly defined	Yes	Yes	Yes
1.8 The assessment of outcome is made blind to exposure status. If the study is retrospective this may not be applicable	No	Yes	Yes
1.9 Where blinding was not possible, there is some recognition that knowledge of exposure status could have influenced the assessment of outcome	Yes	Can’t say	Can’t say
1.10 The method of assessment of exposure is reliable	Yes	Yes	Yes
1.11 Evidence from other sources is used to demonstrate that the method of outcome assessment is valid and reliable	Yes	Yes	Yes
1.12 Exposure level or prognostic factor is assessed more than once	Yes	No	No
1.13 The main potential confounders are identified and taken into account in the design and analysis	Yes	Yes	Yes
1.14 Have confidence intervals been provided	Yes	Yes	Yes
2.1 How well was the study done to minimise the risk of bias or confounding?	++	++	++
2.2 Taking into account clinical considerations, your evaluation of the methodology used, and the statistical power of the study, do you think there is clear evidence of an association between exposure and outcome?	Yes	Yes	Yes
2.3 Are the results of this study directly applicable to the patient group targeted in this guideline?	Yes	Yes	Yes


**Table 4:** Methodology checklist case-control studies (SIGN)^35^

	Helenius et al 2005^16^	Sezer et al, 2011^36^	Keller et al, 2013^21^	Kang et al, 2015^20^	Fabri et al, 2015^12^	Bisanz et al, 2016 ^5^	Mohammed et al 2017^27^	Bautista-Maulano et al, 320^17^	Ziebolz et al, 2018^45^	Schmalz et al, 2018^34^	Białowąs et al, 2019^4^	Enginar et al, 2021^11^	Schmalz et al, 2021^33^	Daltaban et al, 2022^8^
1.1 The study addresses an appropriate and clearly focused question	Yes	Yes	Yes	Yes	Yes	Yes	Yes	Yes	Yes	Yes	Yes	Yes	Yes	Yes
1.2 The cases and controls are taken from comparable populations	No	No	Yes	Yes	Yes	No	No	Yes	Yes	Yes	Yes	No	No	Yes
1.3 The same exclusion criteria are used for both cases and controls	Yes	Yes	Yes	Yes	Yes	No	Yes	Yes	Yes	Yes	Yes	Yes	Yes	Yes
1.4 What percentage of each group (cases and controls) participated in the study?	Case: 100% Control: 100%	Case: 100% Control: 100%	Case: 100% Control: 100%	Case: 41.67% Control: 44.05%	Case: 41.67% Control: 41.67%	Case: 100% Control: 100%	Case: 100% Control: 100%	Case: 100% Control: 100%	Case: 100% Control: 100%	Case: 100% Control: 100%	Case: 40% Control: Cannot say	Case: 100% Control: 100%	Case 100% Control 100%	Case: 100% Control: 100%
1.5 Comparison is made between participants and non-participants to establish their similarities or differences	Can not say	Can not say	Can not say	No	No	Can not say	Can not say	Can not say	Can not say	Can not say	No	Can not say	cannot say	Can not say
1.6 Cases are clearly defined and differentiated from controls	Yes	Yes	Yes	Yes	Yes	Yes	Yes	Yes	Yes	Yes	Yes	Yes	Yes	Yes
1.7 It is clearly established that controls are non-cases	Yes	Yes	Yes	Yes	Yes	Yes	Yes	Yes	Yes	Yes	Yes	Yes	Yes	Yes
1.8 Measures will have been taken to prevent knowledge of primary exposure influencing case ascertainment	Yes	No	No	No	Yes	No	No	Yes	No	No	No	No	No	No
1.9 Exposure status is measured in a standard, valid and reliable way	Yes	Yes	Yes	Yes	Yes	Yes	No	Yes	Yes	Yes	No	Yes	Yes	Yes
1.10 The main potential confounders are identified and taken into account in the design and analysis	No	Yes	Yes	No	Yes	Yes	Yes	Yes	Yes	Yes	Yes	No	Yes	Yes
1.11 Confidence intervals are provided	Yes	Yes	Yes	Yes	Yes	No	No	Yes	No	Yes	No	No	No	Yes
2.1 How well was the study done to minimise the risk of bias or confounding?	++	++	++	+	++	+	+	++	++	++	+	+	+	++
2.2 Taking into account clinical considerations, your evaluation of the methodology used, and the statistical power of the study, do you think there is clear evidence of an association between exposure and outcome?	No	No	Yes	No	Yes	No	No	No	Yes	Yes	No	No	No	No
2.3 Are the results of this study directly applicable to the patient group targeted by this guideline?	Yes	Yes	Yes	Yes	Yes	Yes	Yes	Yes	Yes	Yes	Yes	Yes	Yes	Yes


**Table 5:** Methodology checklist Mendelian randomisation study (STROBE-MR)^39^

Item no.	Section	Checklist item	Present (+)/Absent (–)
1	TITLE and ABSTRACT	Indicate Mendelian randomisation (MR) as the study’s design in the title and/or the abstract if that is a main purpose of the study	+
2	INTRODUCTION Background	Explain the scientific background and rationale for the reported study. What is the exposure? Is a potential causal relationship between exposure and outcome plausible? Justify why MR is a helpful method to address the study question	+
3	INTRODUCTION Objectives	State specific objectives clearly, including pre-specified causal hypotheses (if any). State that MR is a method that, under specific assumptions, intends to estimate causal effects	+
4a	METHODS Study design and data sources	Setting: Describe the study design and the underlying population, if possible. Describe the setting, locations, and relevant dates, including periods of recruitment, exposure, follow-up, and data collection, when available	+
4b	METHODS Study design and data sources	Participants: Give the eligibility criteria, and the sources and methods of selection of participants. Report the sample size, and whether any power or sample size calculations were carried out prior to the main analysis	+
4c	METHODS Study design and data sources	Describe measurement, quality control and selection of genetic variants	+
4d	METHODS Study design and data sources	For each exposure, outcome, and other relevant variables, describe methods of assessment and diagnostic criteria for diseases	+
4e	METHODS Study design and data sources	Provide details of ethics committee approval and participant informed consent, if relevant	+
5	METHODS Assumptions	Explicitly state the three core IV assumptions for the main analysis (relevance, independence and exclusion restriction) as well assumptions for any additional or sensitivity analysis	+
6a	METHODS Statistical methods: main analysis	Describe how quantitative variables were handled in the analyses (ie, scale, units, model)	–
6b	METHODS Statistical methods: main analysis	Describe how genetic variants were handled in the analyses and, if applicable, how their weights were selected	+
6c	METHODS Statistical methods: main analysis	Describe the MR estimator (eg, two-stage least squares, Wald ratio) and related statistics. Detail the included covariates and, in case of two-sample MR, whether the same covariate set was used for adjustment in the two samples	–
6d	METHODS Statistical methods: main analysis	Explain how missing data were addressed	–
6e	METHODS Statistical methods: main analysis	If applicable, indicate how multiple testing was addressed	–
7	METHODS Assessment of assumptions	Describe any methods or prior knowledge used to assess the assumptions or justify their validity	+
8	METHODS Sensitivity analyses and additional analyses	Describe any sensitivity analyses or additional analyses performed (eg, comparison of effect estimates from different approaches, independent replication, bias analytic techniques, validation of instruments, simulations)	+
9a	METHODS Software and pre-registration	Name statistical software and package(s), including version and settings used	+
9b	METHODS Software and pre-registration	State whether the study protocol and details were pre-registered (as well as when and where)	–
10a	RESULTS Descriptive data	Report the numbers of individuals at each stage of included studies and reasons for exclusion. Consider use of a flow diagram	–
10b	RESULTS Descriptive data	Report summary statistics for phenotypic exposure(s), outcome(s), and other relevant variables (eg, means, SDs, proportions)	–
10c	RESULTS Descriptive data	If the data sources include meta-analyses of previous studies, provide the assessments of heterogeneity across these studies	+
10d	RESULTS Descriptive data	For two-sample MR: i. Provide justification of the similarity of the genetic variant-exposure associations between the exposure and outcome samples ii. Provide information on the number of individuals who overlap between the exposure and outcome studies	–
11a	RESULTS Main results	Report the associations between genetic variant and exposure, and between genetic variant and outcome, preferably on an interpretable scale	+
11b	RESULTS Main results	Report MR estimates of the relationship between exposure and outcome, and the measures of uncertainty from the MR analysis, on an interpretable scale, such as odds ratio or relative risk per SD difference	+
11c	RESULTS Main results	If relevant, consider translating estimates of relative risk into absolute risk for a meaningful time period	–
11d	RESULTS Main results	Consider plots to visualise results (eg, forest plot, scatterplot of associations between genetic variants and outcome versus between genetic variants and exposure)	+
12a	RESULTS Assessment of assumptions	Report the assessment of the validity of the assumptions	+
12b	RESULTS Assessment of assumptions	Report any additional statistics (eg, assessments of heterogeneity across genetic variants, such as I^2^, Q statistic or E-value)	+
13a	RESULTS Sensitivity analyses and additional analyses	Report any sensitivity analyses to assess the robustness of the main results to violations of the assumptions	+
13b	RESULTS Sensitivity analyses and additional analyses	Report results from other sensitivity analyses or additional analyses	+
13c	RESULTS Sensitivity analyses and additional analyses	Report any assessment of direction of causal relationship (eg, bidirectional MR)	+
13d	RESULTS Sensitivity analyses and additional analyses	When relevant, report and compare with estimates from non-MR analyses	–
13e	RESULTS Sensitivity analyses and additional analyses	Consider additional plots to visualise results (eg, leave-one-out analyses)	+
14	DISCUSSION Key results	Summarize key results with reference to study objectives	+
15	DISCUSSION Limitations	Discuss limitations of the study, taking into account the validity of the IV assumptions, other sources of potential bias, and imprecision. Discuss both direction and magnitude of any potential bias and any efforts to address them	+
16a	DISCUSSION Interpretation	Meaning: Give a cautious overall interpretation of results in the context of their limitations and in comparison with other studies	+
16b	DISCUSSION Interpretation	Mechanism: Discuss underlying biological mechanisms that could drive a potential causal relationship between the investigated exposure and the outcome, and whether the gene-environment equivalence assumption is reasonable. Use causal language carefully, clarifying that IV estimates may provide causal effects only under certain assumptions	–
16c	DISCUSSION Interpretation	Clinical relevance: Discuss whether the results have clinical or public policy relevance, and to what extent they inform effect sizes of possible interventions	–
17	DISCUSSION Generalisability	Discuss the generalizability of the study results (a) to other populations, (b) across other exposure periods/timings, and (c) across other levels of exposure	–
18	OTHER INFORMATION Funding	Describe sources of funding and the role of funders in the present study and, if applicable, sources of funding for the databases and original study or studies on which the present study is based	+
19	OTHER INFORMATION Data and data sharing	Provide the data used to perform all analyses or report where and how the data can be accessed, and reference these sources in the article. Provide the statistical code needed to reproduce the results in the article, or report whether the code is publicly accessible and if so, where	+
20	OTHER INFORMATION Conflicts of interest	All authors should declare all potential conflicts of interest	+


We assessed the risk of bias using the SIGN and STROBE-MR methodologies.^35,39^ All cohort studies were deemed high quality (Table 3), like the MR study (Table 5). Of the case-control studies, eight were classified as ‘high quality’ and six as ‘acceptable’ (Table 4). No studies were excluded for unacceptable bias.

## DISCUSSION

The relationship between rheumatoid arthritis (RA) and PD is now firmly established, demonstrating a bidirectional interaction.^31^ It’s even suggested that non-surgical periodontal treatment could improve the health status of RA patients.^37^ However, although AS is another rheumatic condition presenting similarities, our study shows that its relationship with PD is more disparate.

Due to significant heterogeneity in study designs (3 cohort,^7,17,43^ 14 case-control,^3–5,8,11,12,16,20,21,27,33,34,36,45^ and 1 MR analysis^14^), conflicting definitions of PD and AS, and disparate protocols, a quantitative meta-analysis could not be conducted. Consequently, a narrative synthesis was conducted to qualitatively evaluate the findings.

Of the 18 studies we examined, over half focused on the prevalence of PD in AS patients. The results are conflicting: two studies showed no increase,^4,21^ one reported a significant decrease after anti-TNF therapy,^3^ and four indicated a significant increase.^20,27,43,45^ Consequently, we cannot conclude with certainty, based on this study, an increase in the prevalence of PD in AS patients, even if a trend seems to emerge. This heterogeneity can be explained by differences in the classifications used across studies. For AS, we identified the use of three distinct classifications: the modified New York criteria for one study,^20^ ASAS classification for two others,^3,4^ and a classification based on clinical symptoms, spinal radiography, or the HLA-B27 test for another.^21^ Furthermore, three studies did not even specify their classification.^27,43,45^ We observed a similar problem for PD classifications; only two of the selected studies used the same classification,^3,45^ and one even relied on patient-reported self-examination.^43^ Such disparities in the definitions of PD and AS influence patient inclusion criteria and, consequently, the results obtained.

Our observations also diverge from the conclusions of Pandey et al, who reported an increased prevalence of PD in AS patients.^29^ We believe this difference is due to the included studies. Despite attempts, we were unable to obtain some complete studies, so we chose to exclude them to avoid relying solely on abstracts. However, a review of these abstracts suggests that some of these non-included studies, had they been integrated, might have influenced our observations toward an increased prevalence.^25,30^

Based on the periodontal parameters, a strict effect on BOP, PPD, CAL and PI could not be conclusively determined. In contrast, we found numerous correlations between the values of these indices and certain AS parameters. BOP correlated with MASES, BASDAI, BASFI, ASQoL, CRP, and BASMI.^8,34^ PPD was associated with BASMI, CRP, and AS duration.^5,36^ As for CAL, it showed a relationship with BASMI, MASES, and AS duration.^8,45^

It’s relevant to note a decrease in periodontal parameters in AS patients undergoing anti-TNF-α treatment.^3,12,17^ However, the treatments taken by all patients are not always specified in the studies. Given this information, it’s plausible that AS treatment could influence periodontal parameters and thus mask a potential link between AS and PD. It has been shown that anti-rheumatic molecules such as anti-TNF-α improve periodontal condition.^44^ Since the molecules used are similar or identical, it’s not contradictory that this would also be the case for AS. It is recalled that TNF-α inhibitors are involved in the underlying inflammatory processes of periodontal disease (PD). The improvement in periodontal parameters observed in patients receiving these molecules may be attributed to the systemic action of the medication rather than a direct improvement in their ankylosing spondylitis (AS). By modulating systemic inflammation, these agents could potentially provide indirect benefits in managing periodontal degradation.^1,41^ However, unlike established adjuncts, TNF-α inhibitors are not recognised as therapeutic adjuvants for PD. Even if they mitigate clinical symptoms through systemic pathways, they do not substitute for mechanical periodontal therapy, which remains essential to treat the local etiological factors of the disease.

This study also suggests an impact on salivary and crevicular fluid composition, although further studies on these subjects are necessary.^8,16^ However, we found no clear link from a microbiological perspective. Two studies showed no change in Pg levels or its antibodies,^4,20^ while another observed a significant decrease.^3^ Nevertheless, a correlation was established between anti-Pg IgG levels and mSASSS as well as BASMI.^20^ This approach seems promising and should be explored in other studies, given that the role of Pg in other rheumatic pathologies, particularly RA, has been examined, suggesting that increased exposure to Pg could influence RA 24.

Inflammatory parameters in AS (IL-6, TNF-α, CRP, ESR) appear to be influenced by PD. While investigations into this link have been limited to just two studies, their findings generally align.^7,20^ This suggests a strong rationale for initiating further research to confirm and expand upon these observations.

The study by Han et al showed no genetic association between PD and AS.^14^ However, we find this research avenue highly relevant, given the significant role of genetics in the development of both pathologies.

This study aligns with existing research indicating that PD is not merely an isolated infection.^18,41^ Indeed, it can be hypothesised that PD influences the systemic inflammatory burden in patients with SA. Furthermore, the correlation between various periodontal parameters and SA activity scores suggests that therapeutic or pharmacological intervention for one condition could serve as an adjuvant therapy for the other. Conversely, treating one pathology may mask or diminish the clinical signs of the other; it is therefore essential to consider the potential synergy between these two diseases and their respective treatments. Recognising the bidirectional link between PD and SA is of significant clinical interest, as it warrants a more integrated, multidisciplinary approach to optimise patient care and treatment outcomes for both conditions.

### Limitations

We propose that the findings of this research contribute to refining the understanding of the relationship between AS and PD. However, we identify several major limitations. One main source of bias is the significant heterogeneity of criteria used to diagnose AS and PD in the reviewed studies. This fundamentally compromises the robustness of the data, creating substantial noise across the entire spectrum of clinical and non-clinical results. This variability is particularly pronounced when authors do not adhere to official classifications and diagnostic criteria, which leads to a proliferation of different criteria. This situation results in considerable heterogeneity of the patient populations included, which could potentially bias the results. Although a large portion of the studies made an effort to use official classifications, the absence of this point in bias assessment scales should be highlighted.

Another significant limitation lies in the exclusion of nine studies for which only abstracts were accessible. This lack of full data may have led to an underestimation of certain links between AS and PD. Conversely, this could also have led to an overestimation. It should be noted that the systematic review by Pandey et al was able to include three of these nine studies, underscoring the importance of accessing full texts.^29^ Despite these limitations, our analysis included a greater number of studies than previous reviews, thereby enriching our knowledge of the potential links between these two pathologies. We notably highlighted the concordance of several clinical parameters between AS and PD, such as BOP, PPD, BASMI, and CRP.

Finally, it is worth noting that our review was able to include, for the first time compared to previous documents, a genetic analysis through the study by Han et al, which represents a unique contribution to the existing literature.^14^

## CONCLUSION

This study sought to identify potential links between PD and AS. While we found no clear relationship regarding prevalence, this may be attributed to factors like limited access to all relevant studies and a lack of standardised diagnostic criteria for both pathologies. Nonetheless, a trend suggesting a possible connection appears to be emerging.

We found only one study investigating a genetic link between these two pathologies. It would be interesting for others to focus on this aspect to confirm or challenge these results.

Considering the inflammatory parameters of AS, it seems there is an increase in patients suffering from both AS and PD compared to those suffering only from AS. This increase could be due to the development of low-grade systemic inflammation in PD patients.

Our study also provides insights into how AS medications affect periodontal health. Specifically, the use of anti-TNF-α agents appears to improve the periodontal parameters we examined. Furthermore, the clinical parameters of both diseases indicate a strong association. We observed a correlation between BOP and five AS-related parameters, whereas PPD and CAL correlated with three AS parameters, two of which were shared.

Considering that periodontal treatments improve key periodontal parameters like BOP, PPD, and CAL, it would be of great interest for future research to investigate the impact of non-surgical periodontal therapy on the clinical and/or inflammatory markers of AS. This leads us to a new hypothesis: could periodontal treatment affect AS? This question remains largely unexplored in the current scientific literature, as our systematic review identified no studies specifically addressing this topic. If such a link were established, periodontal treatment could become a valuable non-pharmacological component in the management of AS, potentially enhancing patients’ quality of life without the added risk of side effects or drug interactions.

### Acknowledgements

The authors would like to acknowledge Dr Martin Brillant, Dr Clara Le Calve and Dr Riffay Moussa Ben for their insightful discussions and technical editing of the manuscript.

The authors are grateful also for the support provided by AI tools, Google Gemini, for its role in the translation of sections from French to English.

#### Funding

No specific funding was received from any bodies in the public, commercial or not-for-profit sectors to carry out the work described in this article.

#### Conflict of interest statement

The authors declare no conflict of interest.
